# Contrasting patterns of 5S rDNA repeats in European and Asian ecotypes of greater duckweed, *Spirodela polyrhiza* (Lemnaceae)

**DOI:** 10.3389/fpls.2024.1378683

**Published:** 2024-04-22

**Authors:** Guimin Chen, Anton Stepanenko, Nikolai Borisjuk

**Affiliations:** ^1^ School of Life Sciences, Huaiyin Normal University, Huai’an, China; ^2^ Leibniz Institute of Plant Genetics and Crop Plant Research (IPK), Gatersleben, Germany; ^3^ Department of Molecular Genetics, Institute of Cell Biology and Genetic Engineering, Kyiv, Ukraine

**Keywords:** 5S ribosomal DNA, rDNA loci, duckweed, *Spirodela polyrhiza*, molecular evolution, genome organization

## Abstract

Ribosomal DNA (rDNA) contains highly conserved, specifically organized sequences encoding ribosomal RNAs (rRNAs) separated by variable non-transcribed intergenic spacers (NTSs) and is abundant in eukaryotic genomes. These characteristics make the rDNA an informative molecular target to study genome organization, molecular evolution, and phylogenetics. In this study, we characterized the 5S rDNA repeats in the greater duckweed *Spiroldela polyrhiza*, a species known for its small size, rapid growth, highly conserved genome organization, and low mutation rate. Sequence analysis of at least 12 individually cloned PCR fragments containing the 5S rDNA units for each of six ecotypes that originated from Europe (Ukraine) and Asia (China) revealed two distinct types of 5S rDNA repeats containing NTSs of different lengths and nucleotide compositions. The shorter 5S rDNA repeat units had a highly homogeneous 400-bp NTS, with few ecotype- or region-specific single-nucleotide polymorphisms (SNPs). The longer 5S rDNA units had NTSs of 1056–1084 bp with characteristic intra- and inter-genomic variants due to specific SNPs and insertions/deletions of 4–15-bp DNA elements. We also detected significant variability in the ratio of short/long 5S rDNA variants between ecotypes of *S. polyrhiza*. The contrasting dynamics of the two types of 5S rDNA units, combined with the unusually low repeat copy number (for plants) in *S. polyrhiza* (46–220 copies per genome), shows that this species could serve as an excellent model for examining the mechanisms of concerted evolution and functional significance of rDNA variability.

## Introduction

**Figure 1:**
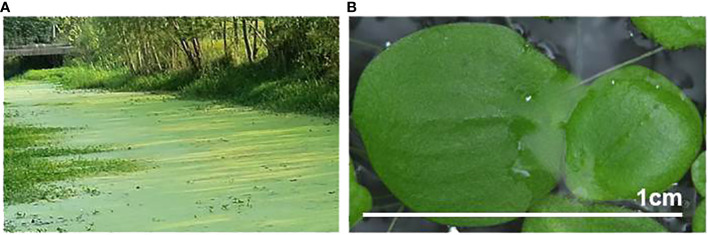
Greater duckweed (*Spirodela polyrhiza*), naturally growing on the surface of a body of water **(A)**, and *in vitro*-cultivated individual fronds **(B)**.

Greater duckweed (*Spirodela polyrhiza*) is a monocotyledonous aquatic plant in the family Lemnaceae (Landolt, 1986). Individual duckweed plants are small with simplified body morphology and grow vigorously, forming colonies of single or mixed species (**Figure 1**). Duckweeds comprise 36 species in five genera (Bog et al., 2019) and are a surprisingly diverse group of plants that have applications in basic research to explore genetic, physiological, and biochemical pathways, as well as various practical applications (Cao et al., 2018; Fourounjian et al., 2020; Acosta et al., 2021; Oláh et al., 2023). For example, duckweed biomass is rich in proteins, carbohydrates, crude fiber, and minerals, making it an attractive source of feed for animals (particularly aquaculture) and humans (Appenroth et al., 2017). Additionally, duckweeds have been widely used for wastewater treatment (Zhou et al., 2018), biosensing (Ziegler et al., 2019), and phytoremediation (Ekperusi et al., 2019; Zhou et al., 2023).

*S. polyrhiza* has the largest body size among duckweeds, with individual fronds of about 7–10 mm in diameter (**Figure 1B**). It is the most cosmopolitan duckweed species, inhabiting five continents (Tippery and Les, 2020), and mostly propagates vegetatively (Landolt, 1986). *S. polyrhiza* has a relatively small genome of ~160 Mb distributed among 20 chromosomes (Hoang et al., 2019). Whole-genome sequencing of *S. polyrhiza* ecotypes from the United States (Wang et al., 2014) and Europe (Michael et al., 2017), followed by parallel genome examination of more than 100 additional *S. polyrhiza* ecotypes sampled around the world (Ho et al., 2019; Xu et al., 2019) showed exceptionally low variation rates.

The ribosomal DNA (rDNA) has become a favorite molecular target in studies related to plant systematics, evolution, and biodiversity and is used as a genome-specific marker in allopolyploids and hybrids (Borisjuk et al., 1988; Stadler et al., 1995; Mahelka et al., 2017; Volkov et al., 2017; Stepanenko et al., 2022). The 35S and 5S rDNA loci are organized in clusters of tandemly repeated units composed of conserved sequences that are transcribed to produce rRNAs; these are separated by diverse intergenic spacers (NTSs), which are not transcribed (Volkov et al., 2003; Hemleben et al., 2021). Its abundance, counted in hundreds to thousands of tandemly repeated copies per genome (Prokopowich et al., 2003; Goffová and Fajkus, 2021), and the diversity of the NTSs, make the rDNA a useful target for genomic studies. However, despite substantial progress in whole-genome sequencing techniques (Michael and VanBuren, 2015; 2020), resolving repetitive sequences, such as the tandemly arranged 5S and 35S rDNA repeats (Lopez et al., 2023), remains a challenge with many repeat sequences missing from annotated plant genomes.

The relatively short, tandemly arranged 5S rDNA repeats composed of conserved sequences encoding the 5S rRNA and highly variable NTSs are especially useful for differentiating closely related species and varieties, and for evaluating genomic diversity and genome molecular evolution in numerous plant taxa (Zanke et al., 1995; Matyácek et al., 2002; Simon et al., 2018; Piredda et al., 2021; Tynkevich et al., 2022; Andreev et al., 2023), including duckweeds (Chen et al., 2021). Genomes of *S. polyrhiza* and *S. intermedia*, the only two representatives of the ancient duckweed genus *Spirodela*, have fewer copies of rDNA genes compared to other plants (Michael et al., 2017; Hoang et al., 2018, 2020). Chromosome characterization by *in situ* hybridization showed that both species have two 5S rDNA loci, a major and minor one (Hoang et al., 2019). Consistent with the low variability of nuclear DNA sequences in *Spirodela*, the rDNA producing the 5S rRNA is also highly conserved, as revealed by conventional sequencing of 5S rDNA units of four ecotypes of *S. polyrhiza* (Borisjuk et al., 2018) and *S. intermedia* (Hoang et al., 2020). Sequence analysis of the 5S rDNA repeats in two ecotypes of *S. intermedia* demonstrated that the two loci have slightly different repeat organization (Hoang et al., 2020). Information on the molecular structure of 5S rRNA genes in *S. polyrhiza* is limited to direct sequencing of specific PCR fragments for four ecotypes isolated in eastern China, showing a single repeat type composed of a conserved 119-bp 5S rRNA gene and a 400-bp NTS with almost no variation in the nucleotide sequence (Borisjuk et al., 2018). Therefore, further studies are needed to shed light on the organization of the 5S rDNA in diverse *S. polyrhiza* ecotypes.

Our recent survey of duckweed biodiversity in Ukraine and China (Chen et al., 2022) revealed *S. polyrhiza* as the dominant duckweed species in both countries. Here, we present data on comparative molecular organization of 5S rDNA repeats in six ecotypes of *S. polyrhiza* isolated in different regions of Ukraine and China. Sequencing of more than hundred cloned PCR fragments covering at least twelve whole 5S rRNA gene units for each ecotype complemented with estimation of the gene copy number, revealed intra- and inter-genome variability and the contrasting evolutional dynamics of two types of 5S rDNA repeat units in the greater duckweed, *S. polyrhiza*.

## Materials and methods

### Plant material

The ecotypes of greater duckweed (*Spirodela polyrhiza*) analyzed in this study were collected in different locations of Ukraine and China from 2016–2019. The duckweed specimens were identified as *S. polyrhiza* by double chloroplast barcoding according to Borisjuk et al. (2015) and their exact geographic origins were listed in our previous publication (Chen et al., 2022). In short, the samples of Ukrainian ecotypes DW30, DW78, and DW100, collected from the local water reservoirs, were sorted according to their morphological characteristics, rinsed with water and directly used for DNA preparation. The three Chinese ecotypes, RDSC2014, RDSC5548, and Ya1, were sterilized and kept under aseptic conditions on agar medium in the collection at Huaiyin Normal University, Hui’an, China (Lam et al., 2020). Since the names of the ecotypes from Ukraine all start with DW, we have added an Sp to the names of Chinese ecotypes for convenience and simplicity thus: Sp2014, Sp5548, and SpYa1.

### Cloning, sequencing and molecular characterization of 5S ribosomal RNA genes

For analysis of *S. polyrhiza* 5S rRNA genes, the specific DNA fragments were amplified by PCR from the same samples of genomic DNA used for the barcoding (Chen et al., 2022). In the standard PCR, we used the primers DW-5S-F and DW-5S-R, which are specific for 5S rDNA (**Supplementary Table 1**), and followed the protocol of Borisjuk et al. (2018). The optimized protocol included the specific Taq polymerase buffer GCI (Takara, Dalian, China), which is designed to amplify GC-rich regions, and an increase in the number of amplification cycles to 40, as applied in (Stepanenko et al., 2022). After separately cutting PCR products of ~0.5 and 1–1.2 kb out of the gel and DNA purification using AxyPrep DNA Gel Extraction Kit (Axygen, United States), the generated fragments were cloned into the vector pMD19 (Takara, Dalian, China) and sequenced using a custom service provided by Sangon Biotech (Shanghai, China). The obtained forward and reversed sequences were assembled and analyzed using the CLC Main Workbench (Version 6.9.2, Qiagen) software. The sequencing data for the S.polyrhiza 5S rDNA clones are available at the NCBI Database (accession numbers OR841168 through OR841270).

The secondary structure of the 5S rRNA was analyzed using CLC Main Workbench (Version 6.9.2, Qiagen) software, based on a modified version the dynamic programming algorithm for free energy minimization (Zuker, 1989).

### Estimation of 5S rDNA copy number

The 5S rRNA gene copies were estimated by qPCR, relating the rates of sample DNA amplification to the standard curve. The standard curve was constructed based on amplification of a dilution series of a specially constructed reference plasmid, pAS-Sp1, which contains sequences of short (Sp-5S-S) and long (Sp-5S-L) 5S rDNA units, a portion of a gene coding for 25S rDNA, and a fragment of a single-copy actin gene, all amplified from the genome of *S. polyrhiza* 9509 (**Supplementary Figure 1**). The reference plasmid pAS-Sp1 was constructed by ligation of the corresponding PCR products into the cloning vector pMD19 (Takara, Dalian, China). The whole sequence of the pAS-Sp1 construct is deposited in GenBank under accession number OR841167.

The rDNA copy number was determined in qPCR reactions prepared with the UltraSybr Mixture (CWBio, Taizhou, China), run on the CFX Connect Real-Time detection system (Bio-Rad, Hercules, USA). For quantification of the 5S rDNA, we used primers specific to the NTS of Sp-5S-S and Sp-5S-L (**Supplementary Table 1**). The concentrations of DNA in the analyzed samples were leveled by corresponding dilutions based on the intensity of DNA bands fluorescence following agarose gel electrophoresis, with further adjustments using NanoDrop 1000 UV-Vis spectrophotometer (Thermo Fisher Scientific, CA, USA). The samples and a tenfold dilution series of the reference plasmid were assayed in the same run. The quality of products was checked by the thermal denaturation cycle. Only the experiments providing a single peak were considered. Three technical replicates were performed for each sample. The obtained data were analyzed using BIO-RAD CFX Manager 3.1 (Hercules, USA) and Microsoft Excel 2016. The total copy number of 5S rRNA units was counted as a sum of both types of NTS.

### Phylogenetic analysis

The maximum-likelihood phylogenetic trees of *S. polyrhiza* NTSs were constructed using the NGPhylogeny web-service (https://ngphylogeny.fr) (Lemoine et al., 2019) with MAFFT Multiple Sequence Alignment (Katoh and Standley, 2013) and PhyML algorithm with SMS (Guindon et al., 2010). Clean sequence alignments were generated utilizing BMGE tools (Criscuolo and Gribaldo, 2010). Bootstrap support was estimated with 100 bootstrap replicates. iTOL (https://itol.embl.de) was used for displaying and annotating the generated phylogenetic trees (Letunic and Bork, 2021).

## Results

### Characterization of 5S rDNA repeats in *S. polyrhiza*

**Figure 2:**
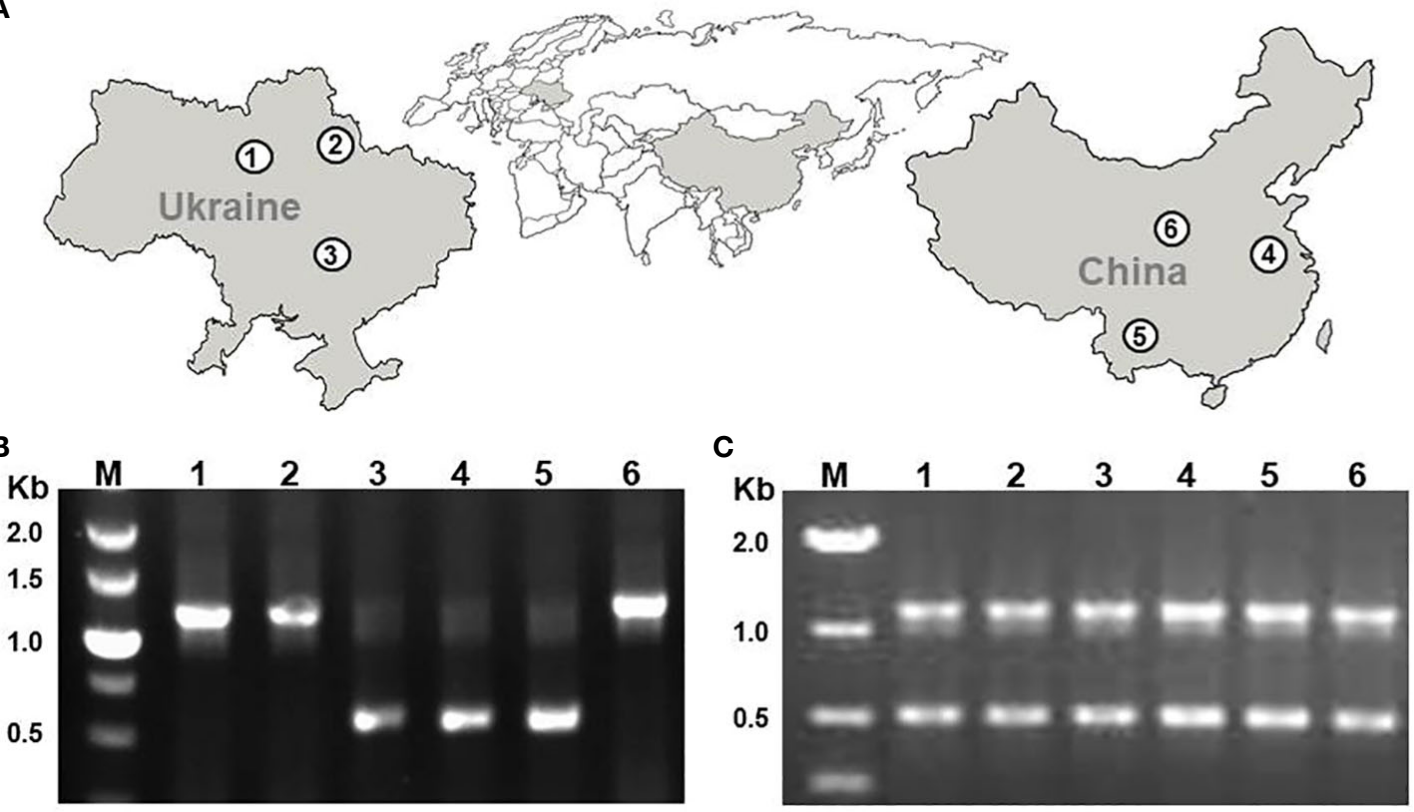
PCR amplification of 5S rRNA genes of six *S. polyrhiza* ecotypes. **(A)** Locations of ecotype sampling; **(B)** PCR products produced by a standard PCR protocol; **(C)** PCR products produced by the optimized PCR protocol. 1, DW30; 2, DW100; 3, DW78; 4, Sp5548; 5, Sp2014; 6, SpYa1. M, DNA marker in kb (1000 base pairs).

In this study, we characterized 5S rDNA in three Ukrainian (DW30, DW100, DW78) and three Chinese (Sp5548, Sp2014, SpYa1) duckweed ecotypes that we previously identified as *S. polyrhiza* by genotyping chloroplast DNA (Chen et al., 2022). The rDNA analysis was performed by sequencing DNA fragments amplified with primers designed to cover the 5S rRNA gene with the NTS in the middle, for each duckweed ecotype. Applying the PCR protocol originally used for amplifying and directly sequencing 5S rDNA fragments in local Chinese strains, including Sp5548 (Borisjuk et al., 2018), resulted in single amplicons with fragment sizes of about 1.1 kb for ecotypes DW30, DW100, and SpYa1, and about 0.5 kb for ecotypes DW78, Sp2014, and Sp5548 (**Figure 2B**). Optimizing the PCR parameters by replacing the standard Taq polymerase with a version adapted for GC-rich DNA regions and increasing the number of reaction cycles resulted in amplification of both the 1.1 kb and 0.5 kb amplicons for each ecotype (**Figure 2C**).

After cutting/purifying the individual PCR fragments from the gel and cloning them, we sequenced at least twelve random individual plasmid clones containing either the small (0.5 kb, Sp-5S-S) or large (1.1 kb, Sp-5S-L) DNA insert for each of the ecotypes.

### 5S rRNA gene sequence

**Figure 3:**
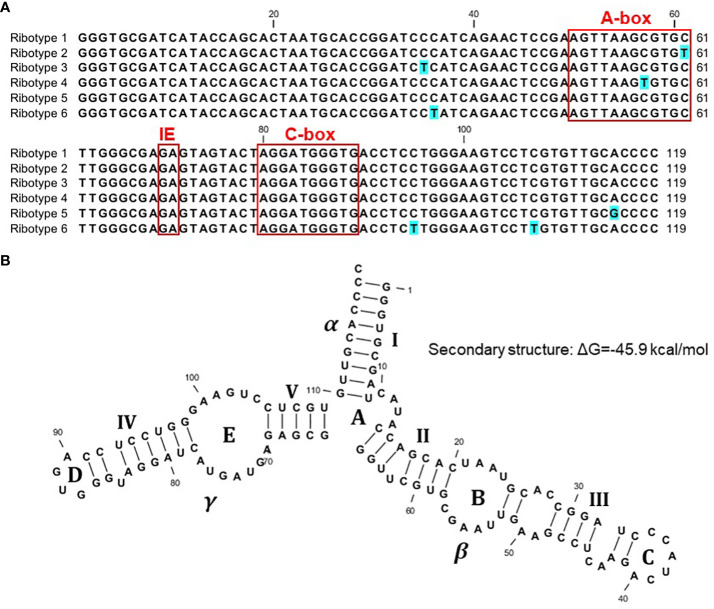
Sequence variants of 5S rDNA identified in the genome of *S. polyrhiza*. **(A)** Nucleotide alignment of the 5S rDNA ribotypes. **(B)** Predicted secondary structure of the full-length 119-nucleotide 5S rRNA, ribotype 1, with three conserved domains (α, β, γ), five stems (I–V), and five loops (A–D).

We started with 103 sequenced clones, including 56 clones containing Sp-5S-S rDNA units, ten of which contained double 5S rDNA units that were apparently visualized as a minor PCR amplicon close to a 1-kb marker in **Figure 2**, and 47 clones containing Sp-5S-L rDNA fragments. From these, we deduced 92 whole sequences coding for 5S rRNA (**Supplementary Figure 1**). We used SNPs in the conserved sequence coding for the 5S rRNA to classify the gene variants into six ribotypes (**Figure 3A**). The dominant ribotype 1 was represented by 74 sequences and ribotypes 2–6 were represented by two to seven sequences. Ribotypes 2–5 differed by single-nucleotide transitions (C/T in ribotypes 2–4 and A/G in ribotype 5) from the dominant ribotype 1. Ribotype 6, represented exclusively by four 5S rRNA gene sequences detected in Sp-5S-L rDNA units of ecotype DW78, had three T/C transitions. All ribotypes contained the internal conserved regulatory elements, such as the A-box, Intermediate Element (IE), and C-Box characteristic of plant 5S rDNA (Cloix et al., 2003). All ribotypes were predicted to form secondary structures with three fingers (domains alpha, beta, gamma), five stems (I–V) and five loops (A–D); these structures are conserved in other plant species (Joachimiak et al., 1990; Garcia et al., 2010; Wicke et al., 2011; Chen et al., 2021; Stepanenko et al., 2022), as shown for ribotype 1 in **Figure 3B**. The changes in secondary structure and free energy values related to the SNP in ribotypes 2–6 are shown in **Supplementary Figure 2**.

### NTS of the Sp-5S-S rDNA repeats

Sequencing of 56 clones with Sp-5S-S rDNA repeats for all six duckweed ecotypes resulted in 66 individual NTS sequences, including 10 clones with dual repeat units for ecotypes DW30, DW78, and Sp2014. Analysis of the NTS structure revealed highly conserved 400-bp sequences with few differences within and between ecotypes. As is common for most plant species, all NTS sequences started with a T-enriched motif, attributed to the conserved site of transcription termination (Cloix et al., 2002). Among the 66 individual NTS sequences, we detected 39 nucleotide substitutions, including 32 nucleotide transitions (10 T/C, 8 C/T, 12 G/A, and 2 A/G), and 7 transversions (3 C/G, 1 G/C, 2 T/A, and 1 C/A). We also detected a single nucleotide insertion in two NTSs of ecotype Sp5548. Two small deletions (one and nine nucleotides) were located in the NTS sequences of ecotypes DW100 and Sp2014, and both deletions occurred in GC-rich regions (**Supplementary Figure 3**).

**Figure 4:**
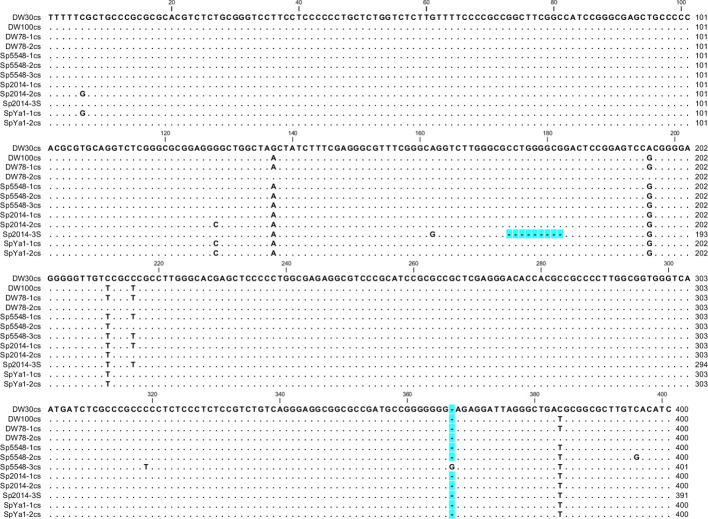
Nucleotide alignment of representative Sp-5S-S NTS consensus sequences of *S. polyrhiza* ecotypes. Gaps are highlighted in blue. The numbers of sequences used for building representative consensus sequences (cs), are DW78-1cs, 8; DW78-2cs, 3; Sp5548-1cs, 9; Sp5548-2cs, 2; Sp5548-3cs, 2; Sp2014-1cs, 10; Sp2014-2cs, 7; SpYa-1cs, 5; SpYa1-2cs, 3.

We divided the NTSs into groups and built consensus sequences for each ecotype. We found only one type of NTS for ecotypes DW30 and DW100, but we found two types for DW78 and SpYa1 and three for Sp2014 and Sp5548. For ecotype Sp2014, 9 and 7 sequences formed two major consensus sequences, and the NTS in Sp2014-3S has a 9-bp deletion (**Figure 4**).

### NTS of the Sp-5S-L type 5S rDNA repeats

In contrast to the mostly uniform length of the *S. polyrhiza* Sp-5S-S rDNA repeats, sequencing of the Sp-5S-L fragments revealed greater length variation between 47 NTSs representing six Ukrainian and Chinese ecotypes. The NTS variants, ranging in length from 1054 to 1086 bp, are defined by the presence/absence of short 13–22-bp sequences in the 5’ end of the NTS at three positions: 15–36, 79–93, and 160–190 (**Supplementary Figure 4**), single insertions/deletions (indels), and by the number of repeated AG bi-nucleotides in the middle of the NTS, at position 605–636. We detected these kinds of variants in different combinations between the ecotypes and among the clones of the same ecotype. Some of the indels were ecotype-specific, such as one nucleotide deletion at position 7 in ecotype Sp78, a 22-bp deletion in ecotype Sp5548 (position 15–36), an insertion of a 15-bp element in ecotype DW78 (position 79–93), and the 31-bp insertion at position 160–190 in Sp5548. By contrast, the NTS with a 13-bp deletion at position 15–27 was shared by three different ecotypes. The rDNA repeat with this deletion is the only type found in Ukrainian ecotype DW78, represented by 7 out of 10 clones in DW30, and by 1 out of 10 clones in the Chinese ecotype Sp2014. We detected 108 substitutions among 47 sequences in the NTS region of the Sp-5S-L rDNA repeats. These included 83 nucleotide transitions (26 T/C, 13 C/T, 19 G/A, and 25 A/G), and 25 transversions (3 C/G, 2 G/C, 4 A/T, 11 T/A, 2 A/C, 1 C/A, and 2 G/T).

In addition to the relatively rare SNPs randomly distributed along the NTS sequences, we detected several SNPs specific for a certain ecotype or shared by a couple of ecotypes. In particular, we detected the most ecotype-specific SNPs in DW78, represented by six nucleotide transitions (positions 55, 334, 379, 471, 675, and 789) and three transversions (positions 191, 238, and 772), followed by ecotype Sp5548 with three specific transitions (positions 347, 414, and 698) and a single C↔A transversion at position 529, and ecotype Sp2014 with a specific T↔C transition at positions 67. Three specific nucleotide transitions (positions 61, 126, and 421) were also common for ecotypes DW78 and Sp5548. The DW78, Sp5548, Sp2014, and SpYa1 ecotypes share an enlarged version of a GA_n_ element consisting of 11–16 GA dinucleotides, contrasting with the GA_n_ element comprising 6–8 GA repeats in ecotypes DW30 and DW100.

**Figure 5:**
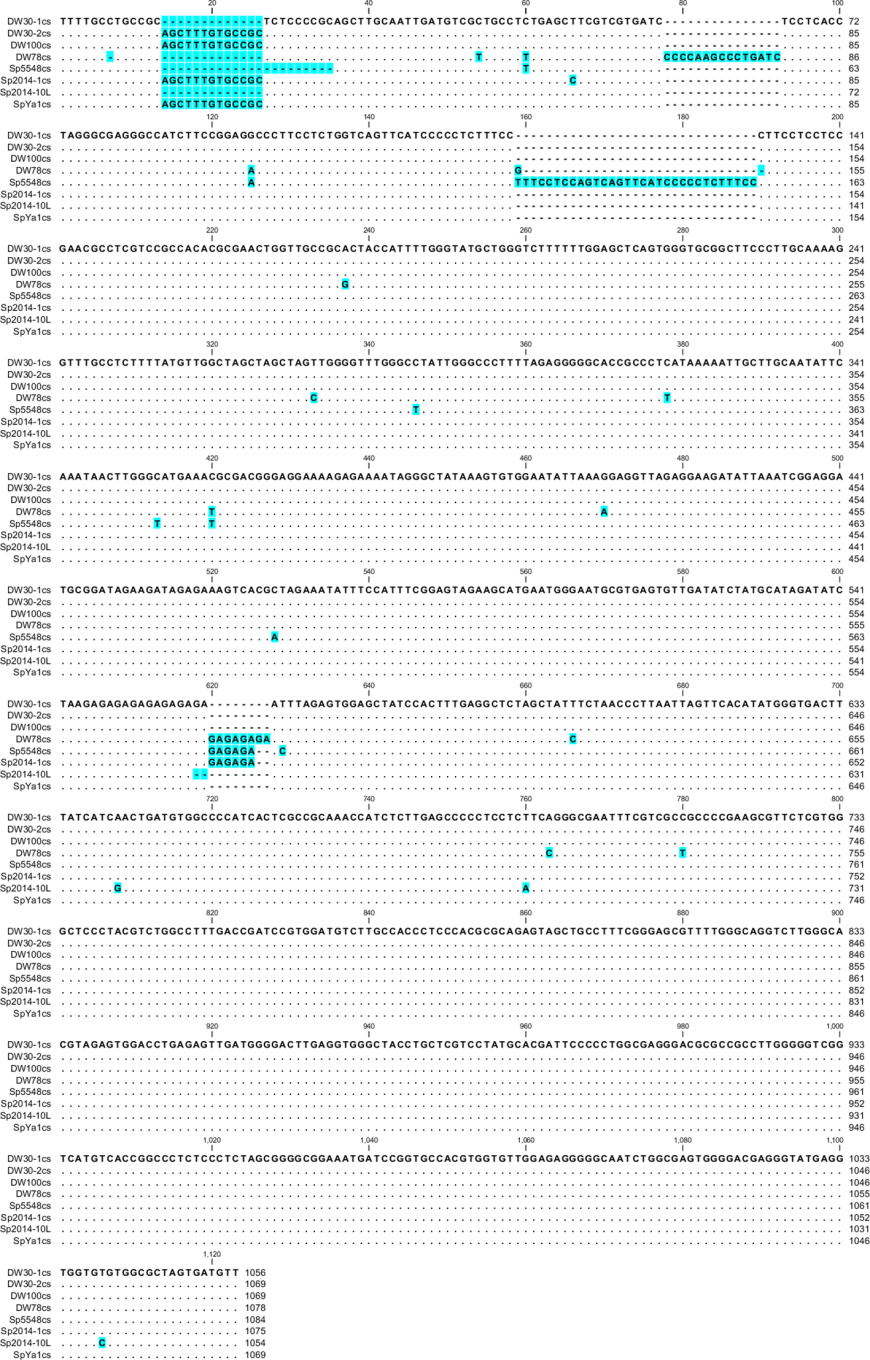
Nucleotide alignment of the consensus Sp-5S-L NTS from *S. polyrhiza*. Gaps and changed residues are highlighted in blue. The number of sequences used for consensus: DW30-1cs, 7; DW30-2cs, 3; Sp2014-1cs, 12.

Based on these data, we divided the NTS into groups and built consensus sequences for each ecotype. We found only one type of NTS for ecotypes DW100, DW78, Sp5548, and SpYa1, but we found two for DW30 and Sp2014 (**Figure 5**). For ecotype Sp2014, 12 sequences formed a major consensus, and the NTS of Sp2014-10L had a 13-bp deletion.

**Figure 6:**
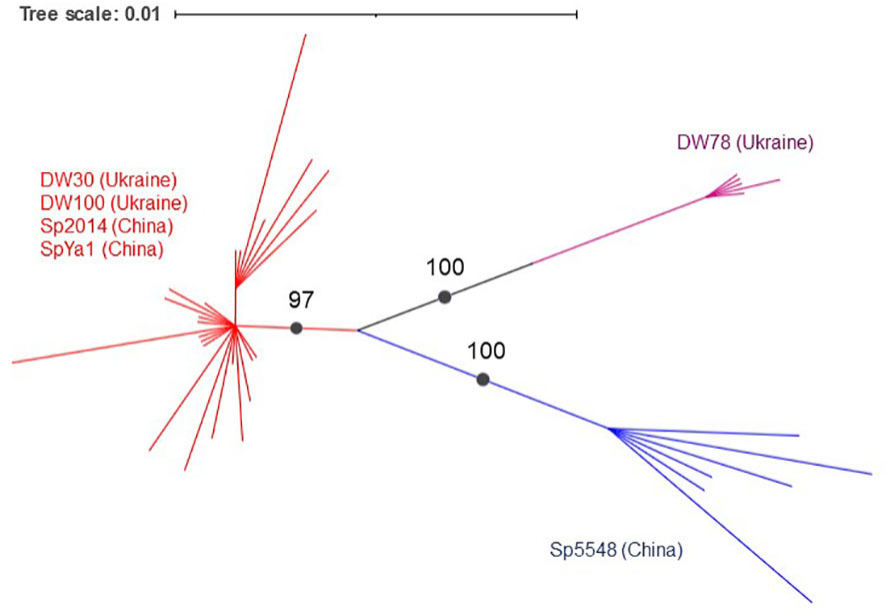
Grouping of duckweed Sp-5S-L NTS sequences. The phylogram shows a maximum likelihood tree obtained using 47 NTS sequences from six duckweed ecotypes. Numbers indicate a bootstrap value for a branch with strong support. Branch lengths represent the expected number of substitutions per nucleotide site. Strongly supported clades are indicated in different colors.

A phylogenetic analysis using the PhyML+SMS algorithm divided Sp-5S-L NTSs into three sub-clusters with strong support (**Figure 6**). The first sub-cluster included all NTSs from ecotype DW78 (Ukraine). The second sub-cluster included the NTS from ecotype Sp5548 (China). The third sub-cluster consisted of all other NTSs.

### Estimation of 5S rDNA copy number and ratio of Sp-5S-L/Sp-5S-S rDNA repeats

The two species in the genus *Spirodela* have been reported to have significantly lower copy numbers of both 35S rDNA (Michael et al., 2017; Hoang et al., 2020) and 5S rDNA genes compared to other plants (Hoang et al., 2018; 2020). In particular, the *S. polyrhiza* strain 9509 contains 73 copies of the 5S rDNA, as estimated by extra-long Oxford Nanopore sequencing (Hoang et al., 2018), and *S. intermedia* strains 8410 and 7747 contain 57 and 70 copies of the 5S rDNA genes, respectively (Hoang et al., 2020), with unequal distribution between the two 5S rDNA loci in both species.

We evaluated the total copy number of 5S rDNA repeats by summing up the number of Sp-5S-S and Sp-5S-L repeat units revealed by quantitative PCR using primers specific for the *S. polyrhiza* 5S rDNA in combination with the primers specific for Sp-5S-S and Sp-5S-L NTSs. A specifically designed DNA construct containing sequences of 5S rRNA genes, Sp-5S-L and Sp-5S-S NTSs, and a unique single copy gene, *Actin*, was used as the internal reference for standardizing the calculations for all analyzed ecotypes (**Supplementary Figure 5**).

The copy number of 5S rRNA genes in the investigated duckweed ecotypes ranged from 46 ± 9 copies in Sp5548 to 220 ± 29 copies in Sp2014 (**Table 1**). The comparative measurement of Sp-5S-L and Sp-5S-S repeats made it possible to divide the studied duckweed ecotypes into two groups, the first one with predominantly Sp-5S-L units and a Sp-5S-L/Sp-5S-S ratio greater than 1 (DW30, ratio 3.03 ± 1.1; DW100, 2.5 ± 0.75; and SpYa1, 1.7 ± 0.4), and the second group with the prevalent 5S rDNA repeats of Sp-5S-S (DW78, ratio 0.16 ± 0.5; Sp2014, 0.26 ± 0.08; and Sp5548, 0.48 ± 0.18).

**Table 1:** Copy number of 5S rRNA genes and the ratio of Sp-5S-L/Sp-5S-S NTSs of six *S. polyrhiza* ecotypes.

Ecotype	Origin	Sp-5S-S	Sp-5S-L	Copy number, Sp-5S-S+ Sp-5S-L	The ratio of NTS types Sp-5S-L/Sp-5S-S
DW30	Ukraine	20 ± 5	61 ± 15	81	3.03 ± 1.1
DW100		30 ± 7	76 ± 14	107	2.5 ± 0.75
DW78		106 ± 16	17 ± 3	123	0.16 ± 0.5
Sp5548	China	31 ± 6	15 ± 3	46	0.48 ± 0.18
Sp2014		178 ± 23	42 ± 9	220	0.26 ± 0.08
SpYa1		46 ± 6	77 ± 11	123	1.7 ± 0.4

## Discussion

Our recent study of duckweed biodiversity in Ukraine and China identified *S. polyrhiza* as the most represented duckweed species in both countries (Chen et al., 2022). In our previous analysis of four Chinese *S. polyrhiza* strains isolated in central-eastern Jiangsu province, direct sequencing of PCR fragments revealed a single unit of 5S rDNA repeats with nearly uniform structure of 119-bp long 5S rRNA genes separated by the 400-bp long NTS (Borisjuk et al., 2018). Genome studies revealed that *S. polyrhiza* and *S. intermedia* contain two 5S rDNA loci located on separate chromosomes (Hoang et al., 2019). Previous research in different plant species suggested that different loci usually contain 5S rDNA repeats with different molecular architecture (Schneeberger et al., 1989; Kellogg and Appels, 1995; Simon et al., 2018; Wang et al., 2023). This was confirmed for two ecotypes of *S. intermedia* where the loci on chromosomes SiChr14 and SiChr15 contain 5S rDNA units composed of identical sequences of 5S rRNA genes separated by NTS sequences, specific for each locus (Hoang et al., 2020). Indeed, despite preferential amplification of a single variant of the 5S rDNA fragments (the short one of about 500 bp for two Chinese ecotypes and one from Ukraine, and the other one of about 1100 bp for the two ecotypes from Ukraine and one from China (**Figure 2B**) using the standard PCR protocol applied earlier (Borisjuk et al., 2018), optimizing the reaction parameters resulted in amplification of both types of 5S rDNA repeats using genomic DNA of each ecotype as template (**Figure 2C**).

The strategy of sequencing multiple individual clones containing 5S rDNA units for each genotype allowed us to gain insight into the intra-genomic variability of gene organization in the analyzed ecotypes. Nucleotide alignments demonstrated high conservation of the 119-bp gene encoding 5S rRNA, with 74 out of 92 obtained gene sequences belonging to a single ribotype. These results confirm our earlier data obtained for two other duckweed species, *S. intermedia* (Hoang et al., 2020) and *Landoltia punctata* (Chen et al., 2021), and the more distantly related aquatic plant *Pistia stratiotes* (Stepanenko et al., 2022), and agree with the general conservation of the 5S rDNA sequence in plants (Park et al., 2000; Hemleben et al., 2021; Tynkevich et al., 2022). However, the 2-D secondary structure model built for the *S. polyrhiza*, following a stem–loop architecture that is typical for plants, showed a characteristic difference in the β domain (**Figure 3**). This difference in the β domain was previously observed in *Landoltia* and *Pistia* (Chen et al., 2021; Stepanenko et al., 2022) and in species from the Asteraceae family (Garcia et al., 2010). In particular, in addition to a smaller loop C compared to the majority of plants, these species have an additional minor loop between loops B and C.

Regardless of this general conservation, we detected several site-specific SNPs represented by C↔T or G↔A transitions in eighteen 5S rRNA genes, grouping the gene sequences into five minor ribotypes in addition to the dominant one. Potentially, the minor ribotypes could be expressed in a tissue- or developmental stage- specific manner, as has been reported for Arabidopsis (*Arabidopsis thaliana*) (Cloix et al., 2002; Layat et al., 2012; Simon et al., 2018). Moreover, the SNPs represented in ribotypes 2 and 4 and located in the regulatory A-box (**Figure 3**) could potentially impact the expression of the genes.

Except for those SNPs, we detected no other variation among the 5S rRNA gene sequences of the *S. polyrhiza* ecotypes, unlike the large number of 5S rRNA pseudogenes reported in the much bigger genomes of the grass *Thinopyrum intermedium* (Mahelka et al, 2013), hexaploid wheat (*Triticum aestivum*) (Sergeeva et al., 2017), and some species in the Solanaceae family (Volkov et al., 2017; Tynkevich et al., 2022). We observed a similar high sequence homogeneity among shorter-length Sp-5S-S variants of *S. polyrhiza* 5S rDNA repeats: only one out of 57 analyzed NTSs of Sp-5S-S with uniform length of 400 bp differed by a 7-bp deletion (clone Sp634-3, **Supplementary Figure 2**). However, in addition to few random nucleotide changes, these NTS sequences contain a number of ecotype-/region-specific SNPs.

Compared to the Sp-5S-S, the longer 5S rDNA units of Sp-5S-L demonstrate significant variability with NTS lengths ranging between 1056 and 1084 bp caused by indels in certain DNA motifs and SNPs in generally conserved core sequences. A significant portion of these changes appeared to be ecotype- or region-specific (**Supplementary Figure 3**). In particular, the highest number of ecotype-specific SNPs was detected in DW78, represented by six nucleotide transitions and three transversions. The long stretch of repeated GA dinucleotides, representing the binding sites for GAGA-binding proteins (GBPs), which play a role in regulating multiple genome functions (Berger and Dubreucq, 2012; Gong et al., 2018; Sahu et al., 2023), is another characteristic feature revealed in the Sp-5S-L NTS sequences of the duckweed 5S rDNA. The DW78, Sp5548, Sp2014, and SpYa1 ecotypes share an enlarged version of the GA_n_ element consisting of 11–16 GA dinucleotides, contrasting with the GA_n_ element in ecotypes DW30 and DW100 containing 6–8 GA repeats.

Alignment of the NTS type-L sequences using phylogenetic tools separated the analyzed ecotypes into two groups (**Figure 5**), one containing NTSs of Ukrainian ecotype DW78 and Chinese ecotype Sp5548, and the other containing the sequences of ecotypes DW30, DW100, Sp2014, and SpYa1. Such a grouping assumes that the ancestors of each group separated prior to their geographic distribution, most probably due to migrating birds. Following their distribution to different geographic locations, the rDNAs in these groups underwent individual clone evolution and occasional hybridization/recombination, which led to the observed complex pattern of the type II NTSs. In that respect, the simultaneous presence of Sp-5S-S NTS variants with SNPs specific for DW30 and the rest of the analyzed ecotypes (**Figure 4**), hints at a possible hybridization event.

Although this study was designed to have a balanced representation of Sp-5S-S and Sp-5S-L 5S rDNA sequences, our estimation of copy number of each type of repeat revealed a highly asymmetric distribution of Sp-5S-S and Sp-5S-L rDNA units among the ecotypes. Thus, based on the ratio of Sp-5S-S/Sp-5S-L units (**Table 1**), the six analyzed ecotypes are clearly divided into two groups with the dominant Sp-5S-S repeats in one group (DW78, Sp2014, and Sp5548) and the Sp-5S-L repeats in another (DW30, DW100, and SpYa1), which agrees with the results of amplification of the 5S rDNA fragments using the non-optimized PCR method (**Figure 2B**). The ratio between the 5S rDNA types among the studied ecotypes widely varied from about 3-fold dominance of Sp-5S-L in ecotype DW30 to more than 6-fold dominance of Sp-5S-S in ecotype DW78, with Sp-5S-L being more prevalent in the Ukrainian ecotypes and Sp-5S-S more common in ecotypes from China. Our data also confirmed the previous estimation (Hoang et al., 2019) that *S. polyrhiza* has one of the lowest copy numbers of 5S rDNA genes among investigated plants, ranging from 46 to 220 copies per genome of Sp5548 and Sp2014 ecotypes, respectively.

We can also assume that, similar to other plants like Arabidopsis (Tutois et al., 2002; Simon et al., 2018), tobacco (*Nicotiana tabacum*) (Fulnecek et al., 2002), fir (*Abies alba*) (Besendorfer et al., 2005), and the duckweed *S. intermedia* (Hoang et al., 2020), the 5S rDNA units of the same type are to certain extent homogenized and arrayed together at one of the two 5S rDNA loci reported for the genome of *S. polyrhiza* (Hoang et al., 2018; 2019). Therefore, the different levels of polymorphism between the 5S rDNA Sp-5S-S and Sp-5S-L arrays are rather locus-specific features, as has been previously noticed for Arabidopsis (Simon et al., 2018) and wheat (Sergeeva et al., 2017). Moreover, the two 5S rDNA loci might be differentially regulated, based on the profound NTS differences of the 5S rDNA arrays at different loci, including regions just upstream of the 5S rRNA genes containing TATA-like boxes, which are implicated in transcription (Cloix et al., 2003).

In summary, our data demonstrate a contrasting speed of molecular evolution between the 5S rDNA units depending on the type of their NTS sequences. Our finding of almost no length variability and low SNP frequency in the short-type NTSs is in agreement with the general low mutation rate in *S. polyrhiza* revealed by whole-genome sequencing of multiple geographically distinct genotypes (Ho et al., 2019; Xu et al., 2019), and the concept of repeated sequence homogenization through the process of concerted evolution (Nei and Rooney, 2005). On other hand, more profound variability of the NTSs of longer Sp-5S-L rDNA units is in contrast with the greater duckweed genome conservation and suggests the influence of some factors suppressing sequence homogenization within the array, as comprehensively discussed in a recent review by Wang et al. (2023). The domination of vegetative propagation might be one of these factors in *S. polyrhiza*, since repeated meiotic crossovers were proposed as the major driving force of DNA repeat homogenization (Smith, 1974; Dover, 1982), with evidence related to rDNA mounting for different eukaryotic species including plants (Pringle et al., 2000; Fehrer et al., 2021; Macháčková et al., 2022). In addition to revealing that *S. polyrhiza* has a very low 5S rDNA copy number compared with most eukaryotes, our data demonstrated intriguing copy number dynamics within the arrays composed of different length variants of 5S rDNA units. Combined, the unique features of greater duckweed’s 5S rDNA that we unveiled in this study represent a promising system for studying basic mechanisms of genome organization, function, and evolution. Much novel information can be gained by reliable long-read sequencing (Hotaling et al., 2023) through the 5S rDNA loci and application of modern genomics for functional characterization of the rDNA variants.

## Data availability statement

The datasets presented in this study can be found in online repositories. The names of the repository/repositories and accession number(s) can be found in the article/**Supplementary Material**.

## Author contributions

GC: Validation, Methodology, Investigation, Writing – review & editing. AS: Writing – original draft, Visualization, Software, Formal analysis, Conceptualization, Validation, Methodology. NB: Writing – review & editing, Supervision, Resources, Project administration, Data curation, Writing – original draft, Conceptualization.
