# Supplementary material for: Contrasting patterns of 5S rDNA repeats in European and Asian ecotypes of greater duckweed, Spirodela polyrhiza (Lemnaceae)
Source: Front Plant Sci. 2024 Apr 22;15:1378683. doi: 10.3389/fpls.2024.1378683 (PMC11070557; doi:10.3389/fpls.2024.1378683)
Supplement: Supplementary file 1 [file DataSheet_1.pdf]

Supplementary materials.

Supplementary Fig. 1S. Nucleotide alignment of 5S rRNA gene sequences of three Ukrainian (DW30, DW78, DW100) and three Chinese (Sp2014, Sp5548, SpYa1) ecotypes of greater duckweed *S. polyrhiza*.

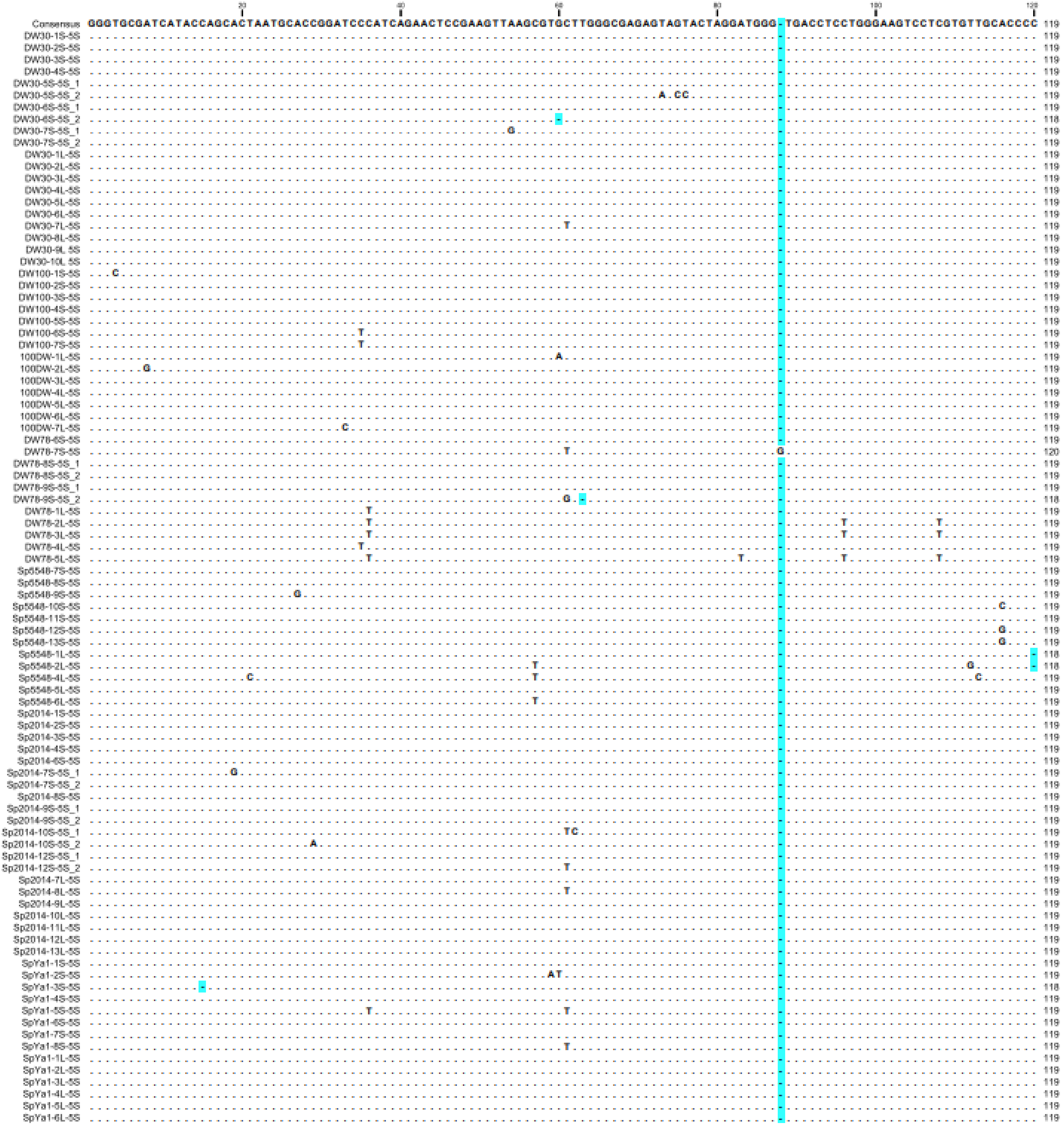

Supplementary Fig. 2S. Predicted secondary structures of five 5S rRNA ribotypes revealed in ecotypes of *Spirodela polyrhiza*. Red boxes indicate nucleotide changes in minor ribotypes 2-6 compared to the dominant ribotype 1.

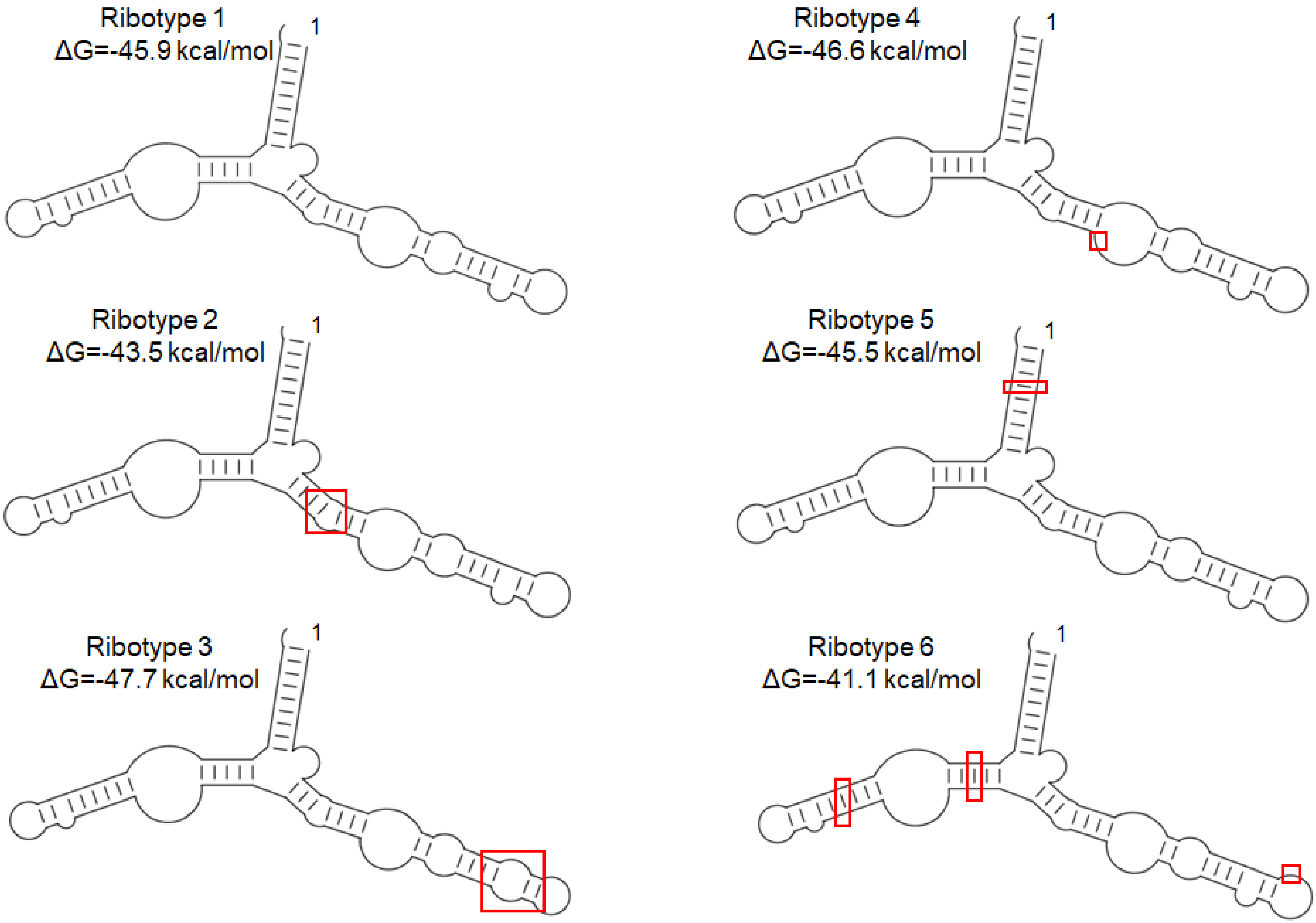

Supplementary Fig. 3S. Nucleotide alignment of type-S NTS sequences of 5S rDNA repeats representing three Ukrainian (DW30, DW78, DW100) and three Chinese (Sp2014, Sp5548, SpYa1) ecotypes of greater duckweed *S. polyrhiza*.

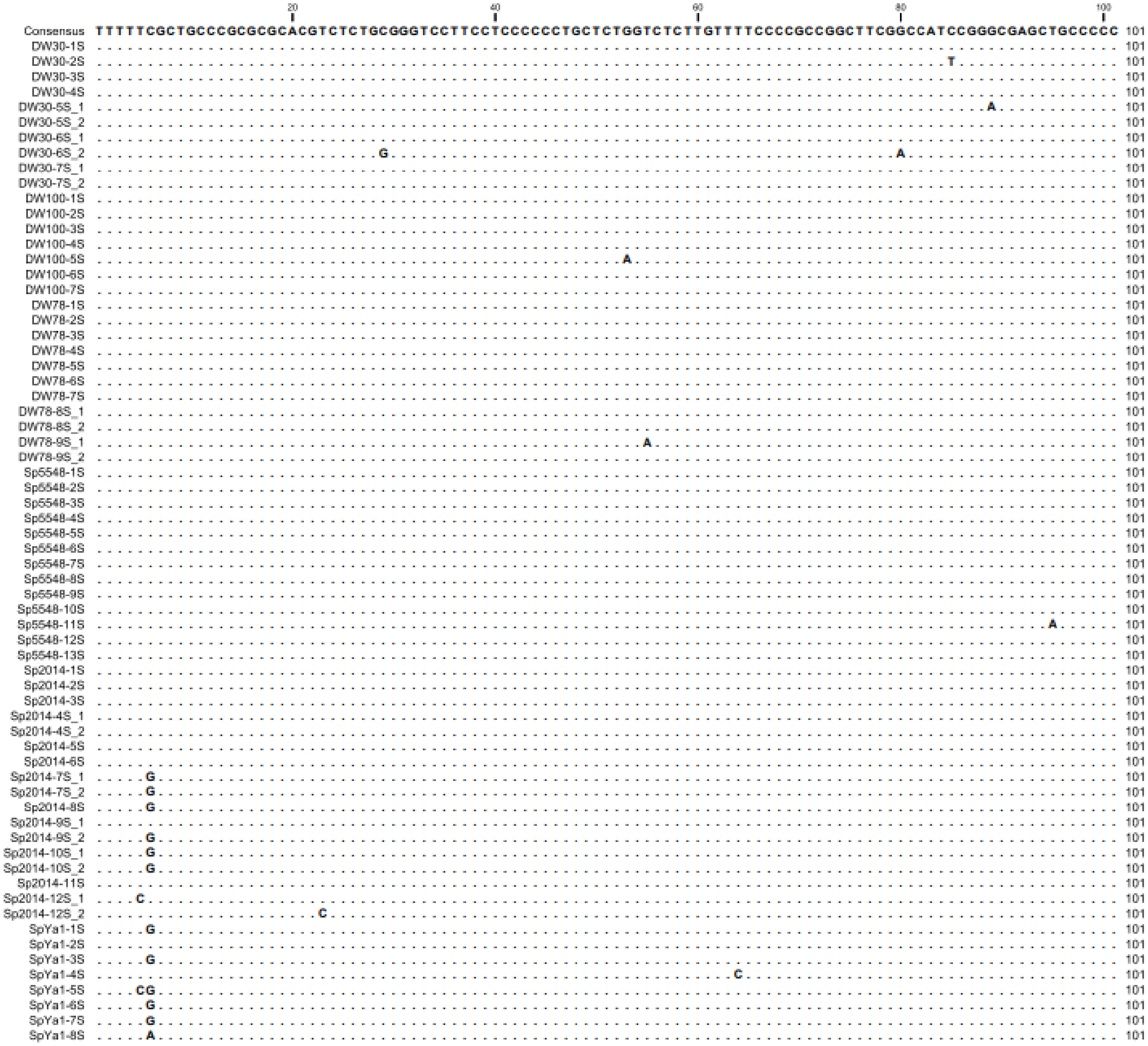

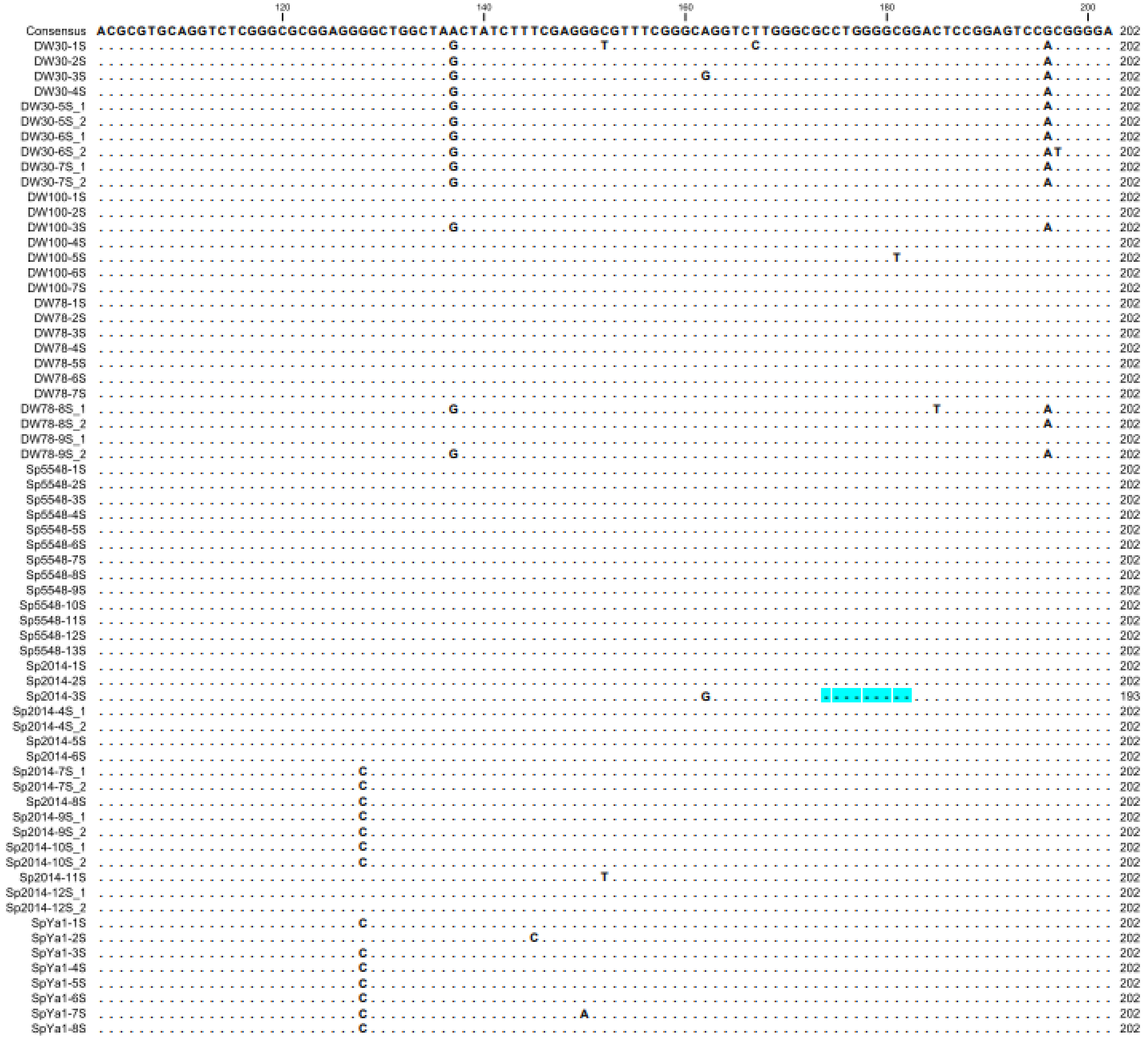

120
140
160
180
200
Consensus ACGCGTGCAGGTCTCGGGCGCGGAGGGGCTGGCTAACTATCTTTCGAGGGCGTTTCGGGCAGGTCTTGGGCGCCTGGGGCGGACTCCGGAGTCCGCGGGGA 202
DW30-1S 202
DW30-2S 202
DW30-3S 202
DW30-4S 202
DW30-5S_1 202
DW30-5S_2 202
DW30-6S_1 202
DW30-6S_2 202
DW30-7S_1 202
DW30-7S_2 202
DW100-1S 202
DW100-2S 202
DW100-3S 202
DW100-4S 202
DW100-5S 202
DW100-6S 202
DW100-7S 202
DW78-1S 202
DW78-2S 202
DW78-3S 202
DW78-4S 202
DW78-5S 202
DW78-6S 202
DW78-7S 202
DW78-8S_1 202
DW78-8S_2 202
DW78-9S_1 202
DW78-9S_2 202
Sp5548-1S 202
Sp5548-2S 202
Sp5548-3S 202
Sp5548-4S 202
Sp5548-5S 202
Sp5548-6S 202
Sp5548-7S 202
Sp5548-8S 202
Sp5548-9S 202
Sp5548-10S 202
Sp5548-11S 202
Sp5548-12S 202
Sp5548-13S 202
Sp2014-1S 202
Sp2014-2S 202
Sp2014-3S 193
Sp2014-4S_1 202
Sp2014-4S_2 202
Sp2014-5S 202
Sp2014-6S 202
Sp2014-7S_1 202
Sp2014-7S_2 202
Sp2014-8S 202
Sp2014-9S_1 202
Sp2014-9S_2 202
Sp2014-10S_1 202
Sp2014-10S_2 202
Sp2014-11S 202
Sp2014-12S_1 202
Sp2014-12S_2 202
SpYa1-1S 202
SpYa1-2S 202
SpYa1-3S 202
SpYa1-4S 202
SpYa1-5S 202
SpYa1-6S 202
SpYa1-7S 202
SpYa1-8S 202

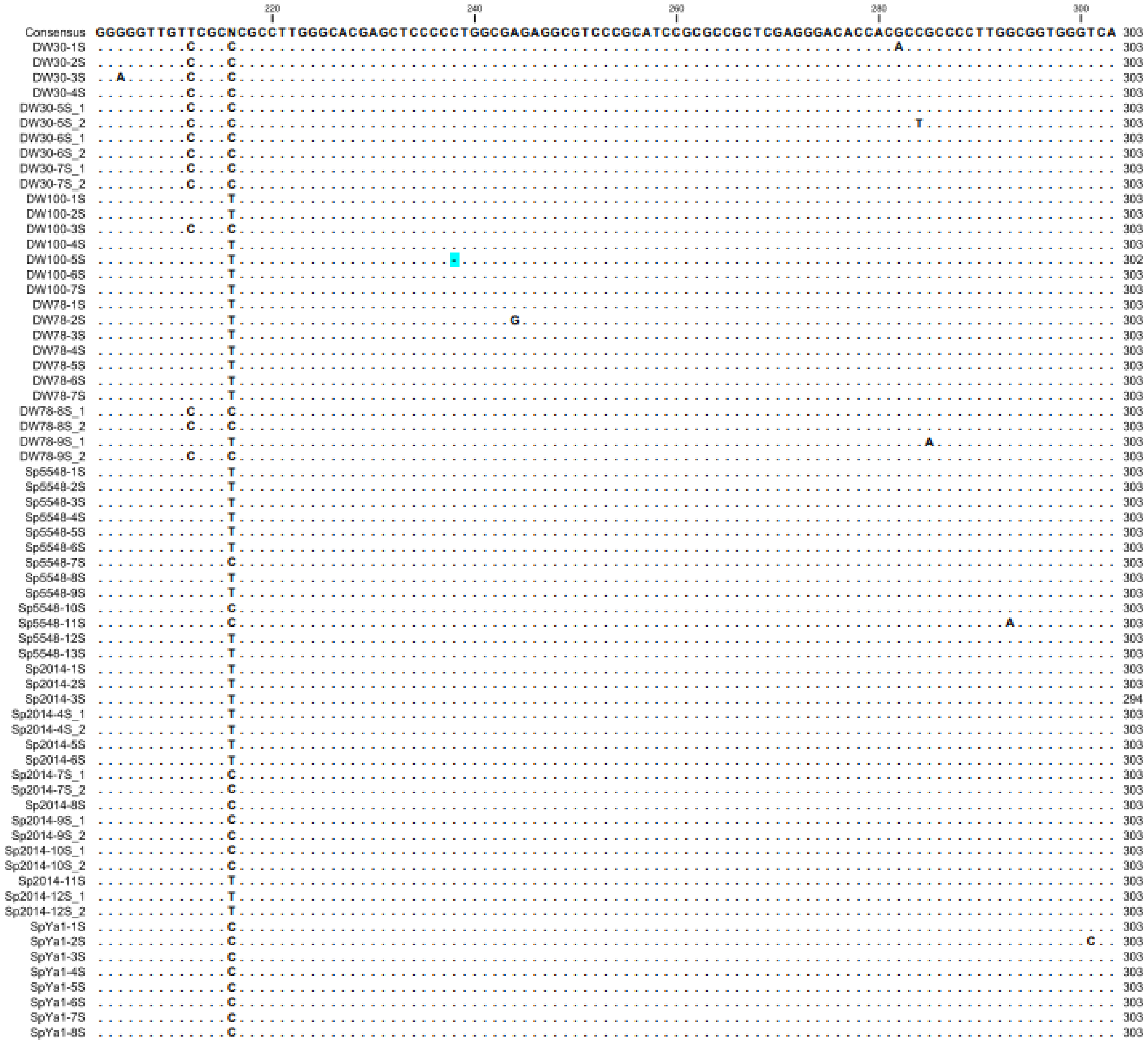

220
240
260
280
300
Consensus GGGGGTTGTTCGCNCGCCTTGGGCACGAGCTCCCCCTGGCGAGAGGCGTCCCGCATCCGCGCCGCTCGAGGGACACCACGCCGCCCCTTGGCGGTGGGTCA 303
DW30-1S 303
DW30-2S 303
DW30-3S 303
DW30-4S 303
DW30-5S_1 303
DW30-5S_2 303
DW30-6S_1 303
DW30-6S_2 303
DW30-7S_1 303
DW30-7S_2 303
DW100-1S 303
DW100-2S 303
DW100-3S 303
DW100-4S 303
DW100-5S 302
DW100-6S 303
DW100-7S 303
DW78-1S 303
DW78-2S 303
DW78-3S 303
DW78-4S 303
DW78-5S 303
DW78-6S 303
DW78-7S 303
DW78-8S_1 303
DW78-8S_2 303
DW78-9S_1 303
DW78-9S_2 303
Sp5548-1S 303
Sp5548-2S 303
Sp5548-3S 303
Sp5548-4S 303
Sp5548-5S 303
Sp5548-6S 303
Sp5548-7S 303
Sp5548-8S 303
Sp5548-9S 303
Sp5548-10S 303
Sp5548-11S 303
Sp5548-12S 303
Sp5548-13S 303
Sp2014-1S 303
Sp2014-2S 303
Sp2014-3S 294
Sp2014-4S_1 303
Sp2014-4S_2 303
Sp2014-5S 303
Sp2014-6S 303
Sp2014-7S_1 303
Sp2014-7S_2 303
Sp2014-8S 303
Sp2014-9S_1 303
Sp2014-9S_2 303
Sp2014-10S_1 303
Sp2014-10S_2 303
Sp2014-11S 303
Sp2014-12S_1 303
Sp2014-12S_2 303
SpYa1-1S 303
SpYa1-2S 303
SpYa1-3S 303
SpYa1-4S 303
SpYa1-5S 303
SpYa1-6S 303
SpYa1-7S 303
SpYa1-8S 303

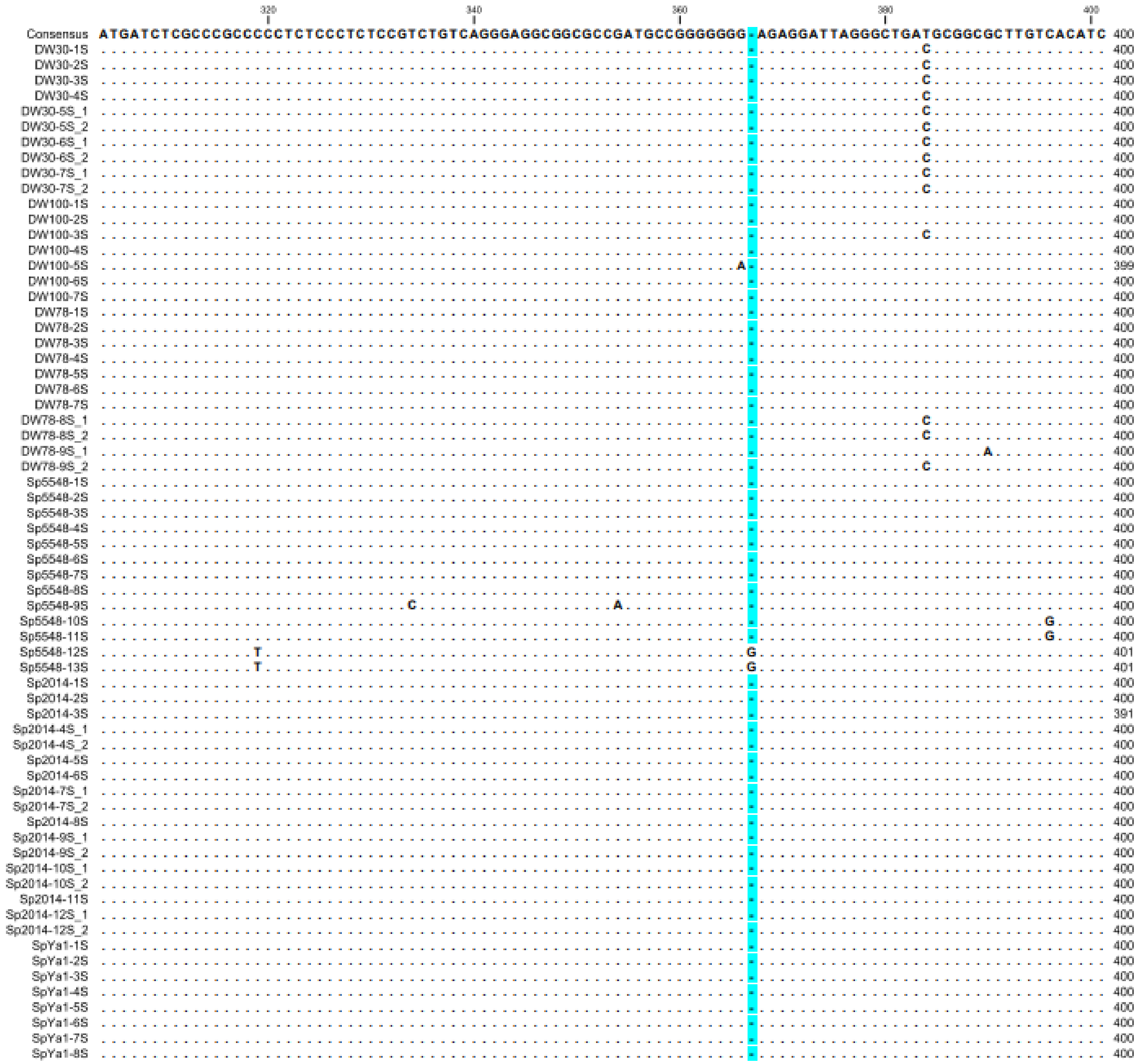
320
340
360
380
400
Consensus ATGATCTCGCCCGCCCCCTCTCCCTCTCCGTCTGTCAGGGAGGCGGCGCCGATGCCGGGGGGG-AGAGGATTAGGGCTGATGCGGCGCTTGTCACATC 400
DW30-1S 400
DW30-2S 400
DW30-3S 400
DW30-4S 400
DW30-5S_1 400
DW30-5S_2 400
DW30-6S_1 400
DW30-6S_2 400
DW30-7S_1 400
DW30-7S_2 400
DW100-1S 400
DW100-2S 400
DW100-3S 400
DW100-4S 400
DW100-5S 399
DW100-6S 400
DW100-7S 400
DW78-1S 400
DW78-2S 400
DW78-3S 400
DW78-4S 400
DW78-5S 400
DW78-6S 400
DW78-7S 400
DW78-8S_1 400
DW78-8S_2 400
DW78-9S_1 400
DW78-9S_2 400
Sp5548-1S 400
Sp5548-2S 400
Sp5548-3S 400
Sp5548-4S 400
Sp5548-5S 400
Sp5548-6S 400
Sp5548-7S 400
Sp5548-8S 400
Sp5548-9S 400
Sp5548-10S 400
Sp5548-11S 400
Sp5548-12S 401
Sp5548-13S 401
Sp2014-1S 400
Sp2014-2S 400
Sp2014-3S 391
Sp2014-4S_1 400
Sp2014-4S_2 400
Sp2014-5S 400
Sp2014-6S 400
Sp2014-7S_1 400
Sp2014-7S_2 400
Sp2014-8S 400
Sp2014-9S_1 400
Sp2014-9S_2 400
Sp2014-10S_1 400
Sp2014-10S_2 400
Sp2014-11S 400
Sp2014-12S_1 400
Sp2014-12S_2 400
SpYa1-1S 400
SpYa1-2S 400
SpYa1-3S 400
SpYa1-4S 400
SpYa1-5S 400
SpYa1-6S 400
SpYa1-7S 400
SpYa1-8S 400

Supplementary Fig. 4S. Nucleotide alignment of type-L NTS sequences of 5S rDNA repeats representing three Ukrainian (DW30, DW78, DW100) and three Chinese (Sp2014, Sp5548, SpYa1) ecotypes of *S. polyrhiza*.

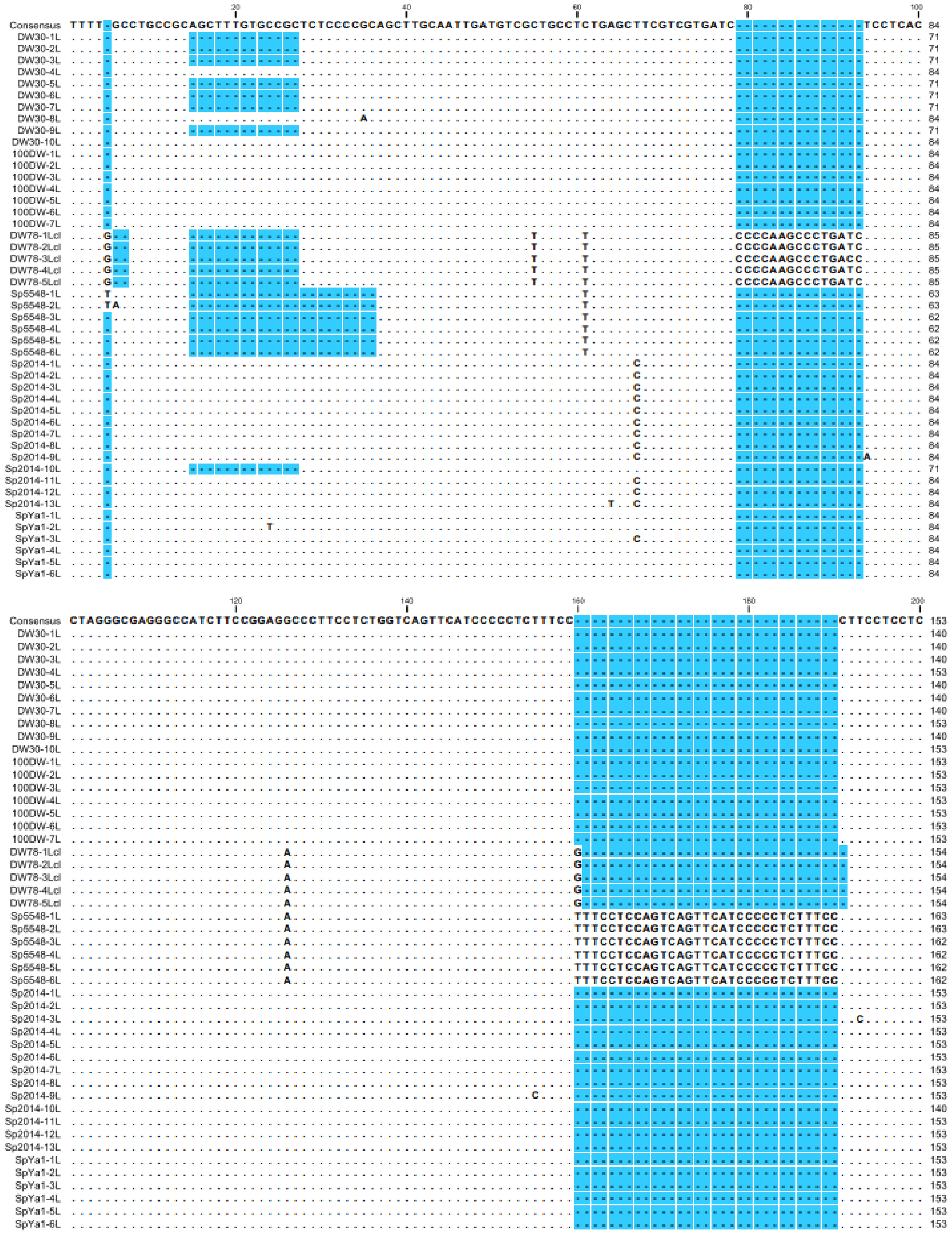

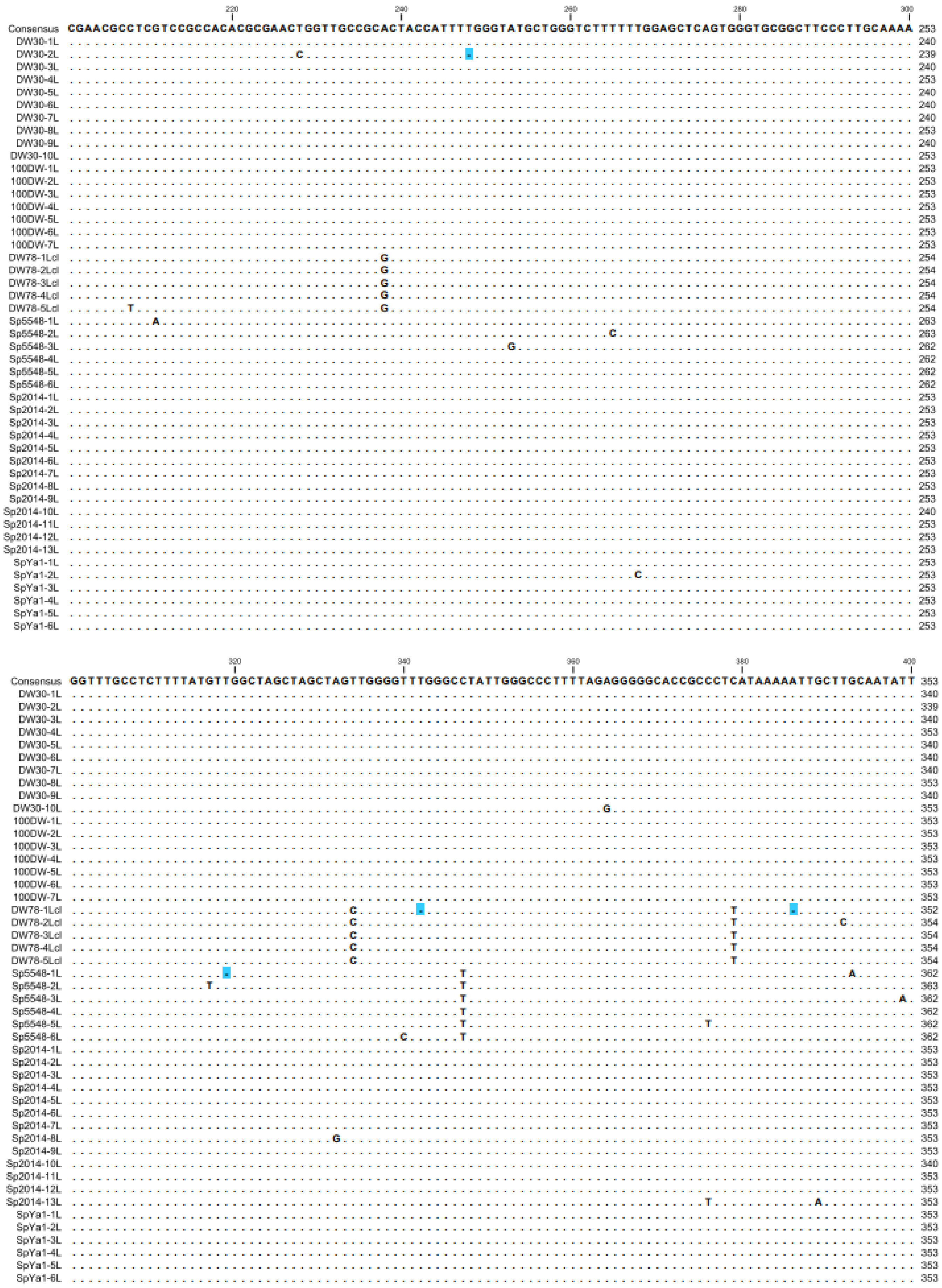

220 240 260 280 300
Consensus CGAACGCCTCGTCCGCCACACGCGAACTGGTTGCCGCACTACCATTTTGGGTATGCTGGGTCTTTTTTGGAGCTCAGTGGGTGCGGCTTCCCTTGCAAAA 253
320 340 360 380 400
Consensus GGTTTGCCTCTTTTATGTTGGCTAGCTAGCTAGTTGGGGTTTGGGCCTATTGGGCCCTTTTAGAGGGGGCACCGCCCTCATAAAAATTGCTTGCAATATT 353

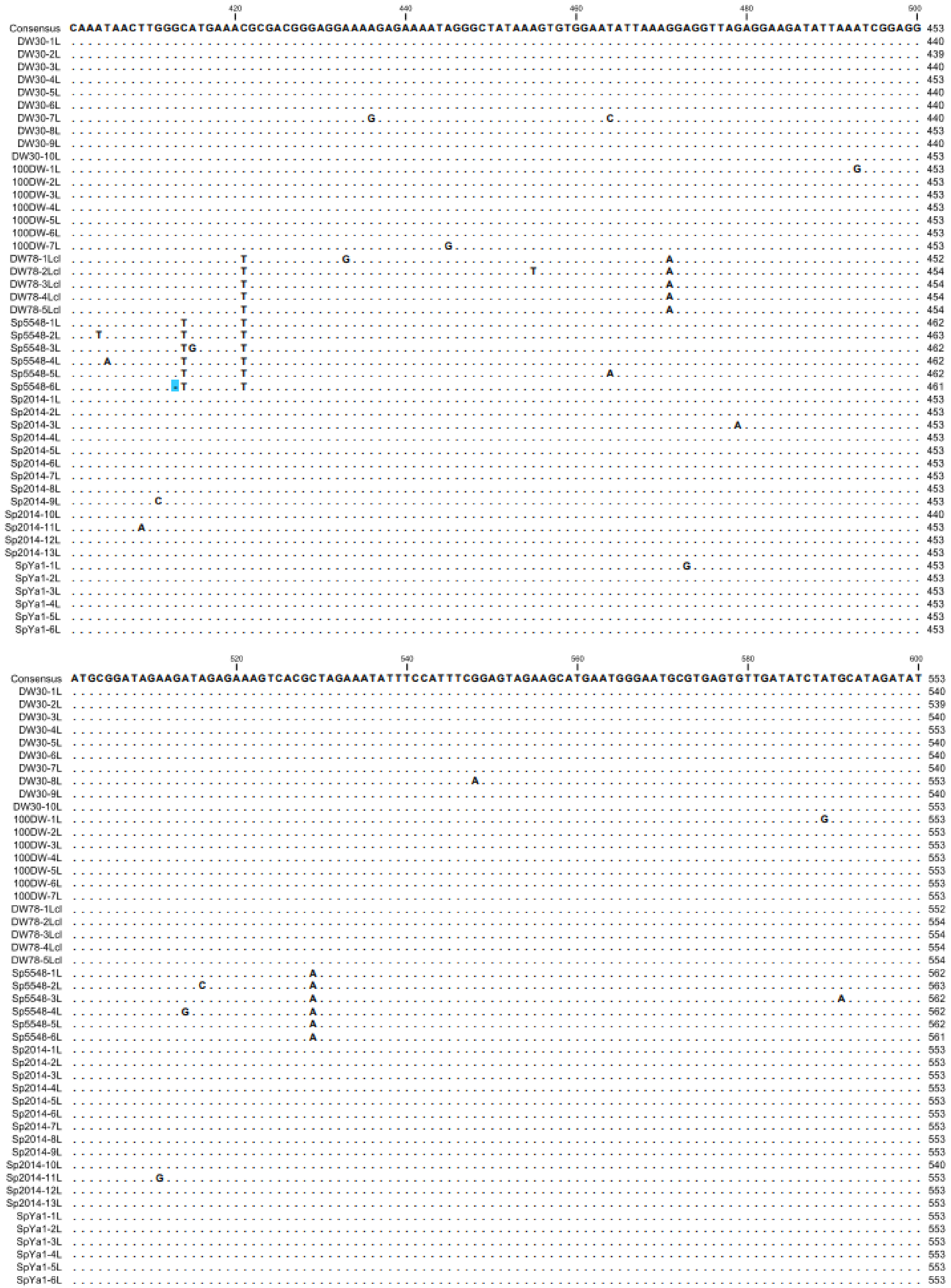

420 440 460 480 500
Consensus CAAATAACTTGGGCATGAAACGCGACGGGAGGAAAAGAGAAAATAGGGCTATAAAGTGTGGAATATTAAAGGAGGTTAGAGGAAGATATTAAATCGGAGG 453
520 540 560 580 600
Consensus ATGCGGATAGAAGATAGAGAAAGTCACGCTAGAAATATTTCCATTTCGGAGTAGAAGCATGAATGGGAATGCGTGAGTGTTGATATCTATGCATAGATAT 553

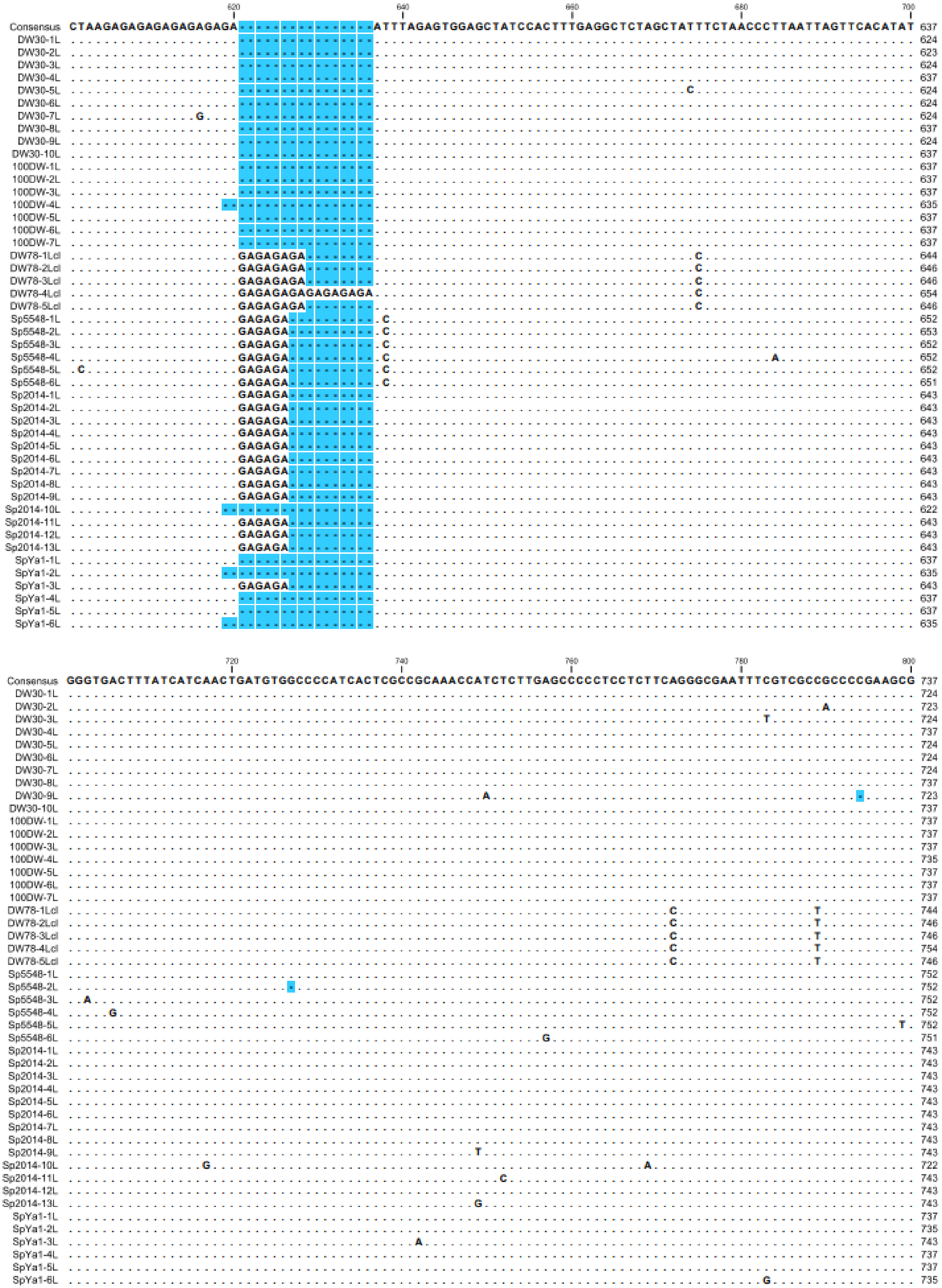
620
640
660
680
700
Consensus CTAAGAGAGAGAGAGAGAGA ATTTAGAGTGGAGCTATCCACTTTGAGGCTCTAGCTATTTCTAACCCTTAATTAGTTCACATAT 637
DW30-1L
DW30-2L
DW30-3L
DW30-4L
DW30-5L
DW30-6L
DW30-7L
DW30-8L
DW30-9L
DW30-10L
100DW-1L
100DW-2L
100DW-3L
100DW-4L
100DW-5L
100DW-6L
100DW-7L
DW78-1Lcl
DW78-2Lcl
DW78-3Lcl
DW78-4Lcl
DW78-5Lcl
Sp5548-1L
Sp5548-2L
Sp5548-3L
Sp5548-4L
Sp5548-5L
Sp5548-6L
Sp2014-1L
Sp2014-2L
Sp2014-3L
Sp2014-4L
Sp2014-5L
Sp2014-6L
Sp2014-7L
Sp2014-8L
Sp2014-9L
Sp2014-10L
Sp2014-11L
Sp2014-12L
Sp2014-13L
SpYa1-1L
SpYa1-2L
SpYa1-3L
SpYa1-4L
SpYa1-5L
SpYa1-6L
720
740
760
780
800
Consensus GGGTGACTTTATCATCAACTGATGTGGCCCCATCACTCGCCGCAAACCATCTCTTGAGCCCCCTCCTCTTCAGGGCGAATTTCGTCGCCGCCCCGAAGCG 737

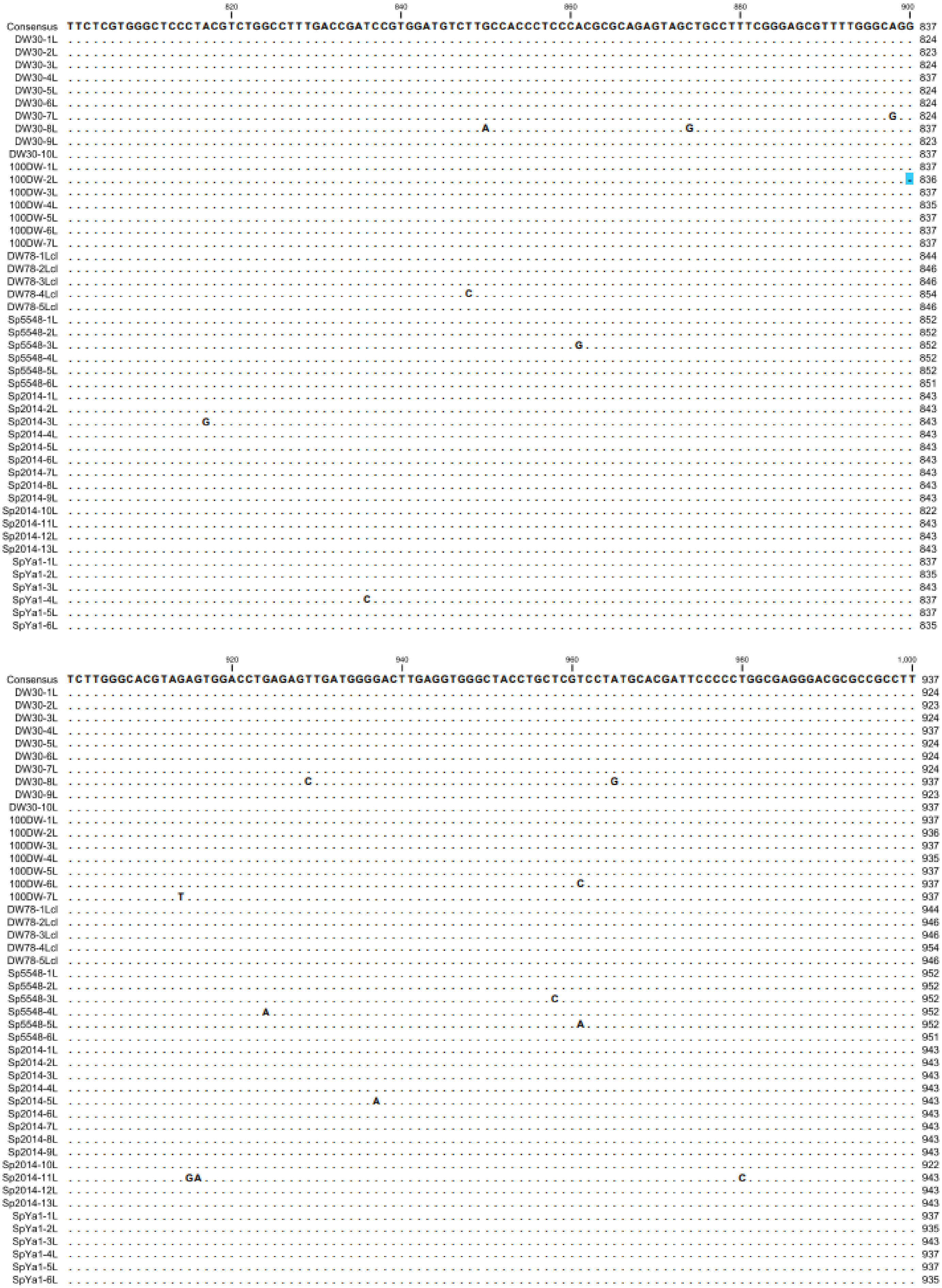
820 840 860 880 900
Consensus TTCTCGTGGGCTCCCTACGTCTGGCCTTTGACCGATCCGTGGATGTCTTGCCACCCTCCCACGCGCAGAGTAGCTGCCTTTCGGGAGCGTTTTGGGCAGG 837
920 940 960 980 1,000
Consensus TCTTGGGCACGTAGAGTGGACCTGAGAGTTGATGGGGACTTGAGGTGGGCTACCTGCTCGTCCTATGCACGATTCCCCCTGGCGAGGGACGCGCCGCCTT 937

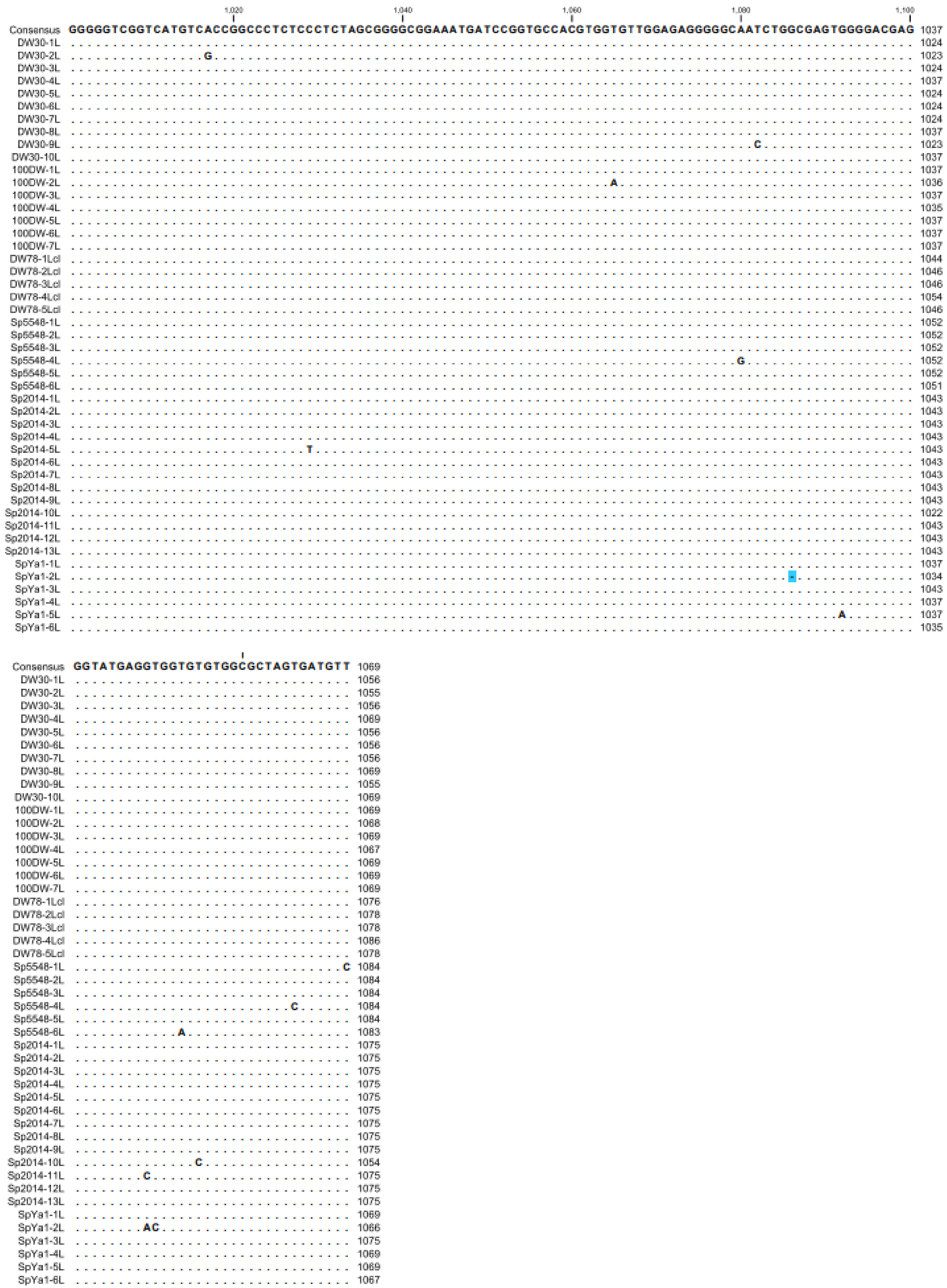
1,020
1,040
1,060
1,080
1,100
Consensus GGGGGTCGGTCATGTCACCGGCCCTCTCCCTCTAGCGGGGCGGAAATGATCCGGTGCCACGTGGTGTTGGAGAGGGGGCAATCTGGCGAGTGGGGACGAG 1037
DW30-1L
DW30-2L
DW30-3L
DW30-4L
DW30-5L
DW30-6L
DW30-7L
DW30-8L
DW30-9L
DW30-10L
100DW-1L
100DW-2L
100DW-3L
100DW-4L
100DW-5L
100DW-6L
100DW-7L
DW78-1Lcl
DW78-2Lcl
DW78-3Lcl
DW78-4Lcl
DW78-5Lcl
Sp5548-1L
Sp5548-2L
Sp5548-3L
Sp5548-4L
Sp5548-5L
Sp5548-6L
Sp2014-1L
Sp2014-2L
Sp2014-3L
Sp2014-4L
Sp2014-5L
Sp2014-6L
Sp2014-7L
Sp2014-8L
Sp2014-9L
Sp2014-10L
Sp2014-11L
Sp2014-12L
Sp2014-13L
SpYa1-1L
SpYa1-2L
SpYa1-3L
SpYa1-4L
SpYa1-5L
SpYa1-6L
Consensus GGTATGAGGTGGTGTGTGGCGCTAGTGATGTT 1069

Supplementary Fig. 5S. Schematic representation of the insert in plasmid pAS-Sp1 used as an internal reference to calculate 5S rDNA copy number of type-S and type-L in *S. polyrhiza* ecotypes. Black arrows indicate locations of the primers used in qPCR reactions.

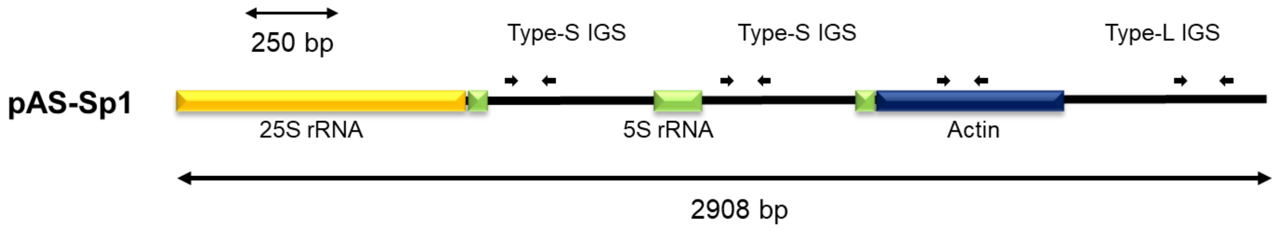

Supplementary Table S1. List of primers used in the study.

Primer ID	Sequence, 5'→3'	Direction	Target	Used for
DW-5S-F	CTTGGGCGAGAGTAGTACTAGG	Forward	5S rDNA	5S rDNA repeats cloning
DW-5S-R	CACGCTTAACTTCGGAGTTCTG	Reverse		5S rDNA repeats cloning
5SIGS-SF1	CCCCTGCTCTGGTCTCTTGT	Forward	Type-S NTS	Copy number estimation
5SIGS-SR1	CCCAAGACCTGCCCGAAAC	Reverse		Copy number estimation
5SIGS-LF1	CCTCTGGTCAGTTCATCCCC	Forward	Type-L NTS	Copy number estimation
5SIGS-LR1	CAATAGGCCCAAACCCCA	Reverse		Copy number estimation
ActinF1	TGTTTTCCCAAGTATCGTC	Forward	Actin	Copy number estimation
ActinR1	TCCCAGTTGGTGACGATT	Reverse		Copy number estimation
